# Brain Structure and Functional Connectivity Correlate with Psychosocial Development in Contemplative Practitioners and Controls

**DOI:** 10.3390/brainsci11060728

**Published:** 2021-05-30

**Authors:** Omar Singleton, Max Newlon, Andres Fossas, Beena Sharma, Susanne R. Cook-Greuter, Sara W. Lazar

**Affiliations:** 1 Athinoula A. Martinos Center for Biomedical Imaging, Massachusetts General Hospital, Charlestown, MA 02114, USA; omars@princeton.edu (O.S.); max.newlon@brainco.tech (M.N.); andres900@gmail.com (A.F.); 2 Vertical Development Academy, Woodside, CA 94062, USA; beena@verticaldevelopment.com; 3 Cook-Greuter and Associates, Wayland, MA 01778, USA; susanne@cook-greuter.com

**Keywords:** ego development, posterior cingulate cortex, resting state, functional connectivity, meditation, self, maturity, default mode network

## Abstract

Jane Loevinger’s theory of adult development, termed ego development (1966) and more recently maturity development, provides a useful framework for understanding the development of the self throughout the lifespan. However, few studies have investigated its neural correlates. In the present study, we use structural and functional magnetic resonance imaging (MRI) to investigate the neural correlates of maturity development in contemplative practitioners and controls. Since traits possessed by individuals with higher levels of maturity development are similar to those attributed to individuals at advanced stages of contemplative practice, we chose to investigate levels of maturity development in meditation practitioners as well as matched controls. We used the Maturity Assessment Profile (MAP) to measure maturity development in a mixed sample of participants composed of 14 long-term meditators, 16 long-term yoga practitioners, and 16 demographically matched controls. We investigated the relationship between contemplative practice and maturity development with behavioral, seed-based resting state functional connectivity, and cortical thickness analyses. The results of this study indicate that contemplative practitioners possess higher maturity development compared to a matched control group, and in addition, maturity development correlates with cortical thickness in the posterior cingulate. Furthermore, we identify a brain network implicated in theory of mind, narrative, and self-referential processing, comprising the posterior cingulate cortex, dorsomedial prefrontal cortex, temporoparietal junction, and inferior frontal cortex, as a primary neural correlate.

## 1. Introduction

Jane Loevinger’s theory of ego development [1] posits that the ‘self’ undergoes a series of systematic and hierarchical changes throughout the lifespan, beginning at birth. Each successive iteration results in a more complex psychological construction of one’s relationship to the self and to the world at large. Cook-Greuter (1999) [2] contributed an important expansion to the theory by dividing the highest stage of ego development into two distinct stages as well as expanding on and differentiating the already extant stages and tiers [3,4]. The shift from one stage to the next represents a significant qualitative and quantitative transformation in the way an individual apprehends reality and makes sense of experiences [2]. The term “ego” was informally described by Loevinger as a “frame of reference” or “lens” through which a person perceives their experience of the world and of themselves [5]. Thus, the term “ego development” implies that the frame of reference can change in important ways, giving rise to significant intrapersonal and interpersonal differences in the process. Indeed, as the ego develops, the individual becomes less dominated by impulses; gains the capacity to view themselves, others, and the world in more complex ways; and tolerates greater degrees of uncertainty and ambiguity [2]. 

Loevinger’s theory and its measurement tool, the Washington University Sentence Completion Test (WUSCT), which uses written narrative samples to assess ego development, have been well-validated in the literature [1,6,7,8,9,10,11,12,13,14]. The version of this measurement tool that we use herein is the Maturity Assessment Profile (MAP), which is the expanded version of the WUSCT [4]. The term “ego development” is often used to refer to this process of lifespan development; since this process describes the development of maturity throughout the lifespan, “maturity development” is sometimes used to describe it and will be used interchangeably hereafter. Other studies reveal relationships between ego development and a myriad of psychological factors, including openness to experience [15,16], wisdom [17], impulsiveness [18], emotional security and intimacy [19], and authoritarianism [20]. For a review, see Gilmore and Durkin (2001) [6]. Although ego development and intelligence have some overlap, several studies have shown that the constructs are empirically and conceptually distinct [5,21]. Although well-validated and empirically supported, the body of literature utilizing this tool has been relatively constrained by the amount of time to administer the assessment as well as the need for highly trained evaluators to score the results. Modern computational techniques may hold promise for alleviating these difficulties and revitalizing this area of research [22].

Importantly, transition through these stages is a natural part of development, with high inter-individual variability in the rate of development. Development often slows down or stalls for most adults during the conventional stages [2]. Eastern contemplative traditions provide a different perspective on ego development, namely that ‘higher’ states of development can be achieved through extensive practice of meditative techniques [23,24]. The highest stages of development described by Western psychological constructs share many similarities to descriptions of highly experienced meditation practitioners. However, there has been a paucity of studies examining the relationship between contemplative practice and ego development [25]. In order to gain preliminary evidence supporting the hypothesis that meditative practices might help catalyze maturity development, the MAP was administered as part of a larger study of the impact of two contemplative practices on cognition [26,27]. 

Neuroscientific research has begun to link constructs related to self-related processes to functional and neuroanatomical findings. In particular, a network of brain regions termed the default mode network (DMN) has been consistently implicated in self-related processes in functional magnetic resonance imaging (fMRI) studies [28,29,30]. The DMN is involved in internally oriented cognition, including daydreaming [31], narrative [32], mind-wandering [33,34], autobiographical memory [35,36], and language [37]. The DMN has also been implicated in numerous psychiatric and neurological disorders involving disruptions in self, including autism, Alzheimer’s, schizophrenia, and affective disorders; for a review, see Broyd et al. (2009) [38]. 

Despite considerable interest in the neural basis of self-related processes, few neuroimaging studies have attempted to ground the lifespan developmental theories in brain structure and function, and none to our knowledge using structural and functional MRI [39]. Given that higher stages of ego development are associated with greater degrees of self-autonomy, effective perspective-taking, self-efficacy, and meaning-making, and since DMN structure and function are related to these cognitive functions, we hypothesized that individual differences in DMN structure and resting state functional connectivity would correlate with ego development.

Mindfulness has become a popular area of research in recent years, resulting in numerous studies investigating the behavioral, physiological, and neurological effects of meditation [40,41,42]. In addition, studies have demonstrated that mindfulness practices may induce changes in self-related processes, including self-referential processing [43], self-efficacy [44], and wellbeing [45]. While the goal of contemplative practice is to transcend the sense of self, many traditions describe a gradual development in the sense of self through various stages. However, this has not been the focus of the literature up to this point, despite some early studies in this direction [25,46]. The present study recruited practitioners of Vipassana meditation and practitioners of Kripalu yoga [47,48]. While the origins of the two contemplative practices differ, since Vipassana meditation and Kripalu yoga are rooted in Buddhist [49] and Indian philosophy [50,51], respectively, both practices cultivate insight into the nature of experience, the self, and universal issues. While both Kripalu yoga and Vipassana meditation utilize meditation techniques, Kripalu yoga also includes physical postures (asana) as well as breathing techniques (pranayama). One goal of the larger study [26,27] was to determine neural similarities and differences associated with yoga and meditation. Thus, a secondary goal of the current study was to also investigate differences in ego development between practitioners of these two contemplative traditions. 

Cross-sectional MRI studies have indicated that the regular practice of meditation is associated with differences in brain structure and function [52,53]. Experienced meditators possess increased resting state functional connectivity within the DMN [54,55] as well as reduced medial prefrontal cortex (MPFC) and posterior cingulate cortex (PCC) activity during meditation [56]. Longitudinal studies of MRI have demonstrated that meditation induces structural and functional changes in the brain [57,58], and in particular, increased gray matter density in the PCC and supramarginal gyrus (SMG) was found following an 8-week mindfulness training program [59]. Overall, the DMN, which plays a vital role in not just self-referential processing but also conscious experience itself, can be influenced by contemplative techniques intended to affect change in perception of the self and its relation to the external world.

In the study presented herein, we investigate the relationship between ego development, contemplative practice, and brain structure and function using structural MRI and fMRI, specifically cortical thickness measures and resting state functional connectivity, respectively. We focused our hypotheses on the DMN, since this network has been consistently demonstrated to be integral to processes and perceptions related to the self. Ego development, as assessed by the MAP, is a measure of the complexity of how the self is perceived in relation to the other. Hence, we expected higher scores on the MAP to correlate with cortical thickness and resting state functional connectivity differences in the DMN. In particular, we hypothesized that cortical thickness of the PCC, which is the primary hub of the DMN, would correlate significantly and positively with scores on the MAP. Likewise, functional connectivity within the DMN would correlate with MAP scores. Finally, we hypothesized that MAP scores would be higher in subjects who meditate or practice yoga than in non-meditating matched control subjects. 

## 2. Materials and Methods 

Forty-seven participants, 16 yoga practitioners, 14 meditation practitioners, and 17 controls were recruited. Due to missing data, 47 were included in the behavioral analysis, 46 were included in the structural analyses, and 45 were included in the functional connectivity analyses. The three groups were matched for age, sex, education, race, and handedness, and all participants were free of dementia as assessed with the Mini-Mental State Examination [60] (see Table 1 for demographics). These subjects are a subset of a group reported in Gard et al. (2014) [26]. Yoga practitioners were trained in the Kripalu Yoga tradition [47] and had an average of 13,534 ± 9950 hours or 18.3 ± 9.6 years of yoga experience. Meditators were trained in Vipassana meditation [48] and had an average of 7774 ± 6237 hours or 15.8 ± 7.6 years of meditation experience. Controls had little or no experience with either yoga or meditation. Participants completed brain scans, questionnaires, and a projective assessment on the same day. Participants provided written informed consent and were compensated with $100 for participating. The study was approved by the Partners Human Research Committee, Massachusetts General Hospital (protocol 2005P001392).

Table 1: Demographics and other variables: Raven’s Advanced Progressive Matrices (RAPM); American version of the National Adult Reading Test (AMNART); Total Weighted Score (TWS) (on Sentence Completion Test); Mayer–Salovey–Caruso Emotional Intelligence Test (MSCEIT); Self-Compassion Scale (SCS): Self-Kindness Items (SK), Self-Judgment Items (SJ), Common Humanity Items (CH), Isolation Items (II), Mindfulness Items (MI), and Over-Identified Items (OI); Five Factor Mindfulness Questionnaire (FFMQ): Observing Items (OI), Describing Items (DI), Acting with Awareness Items (AWA), Non-Judging of Inner Experience Items (NJ), and Nonreactivity to Inner Experience Items (NRI). A chi-square test was used to test for group differences in sex; ANOVAs were used for all other variables, except both amounts of practice where pair-wise tests are used, since controls have no experience. The *p*-values in bold indicate *p* < 0.05.

### 2.1. Ego Development 

The Maturity Assessment Profile (MAP), based on the revised Washington University Sentence Completion Test (WUSCT), was used to assess ego development stage [4]. It consists of 36 unfinished sentences for the participant to complete in any way they wish; for example, “When people are helpless…” The MAP is based on the assumption that language reveals the underlying structure of how one is making sense and meaning of experience. Tests were scored by a blinded trained expert scorer according to the revised guidelines [2]. The measure has consistently shown high inter-rater reliability and internal consistency across the literature [6]. The total weighted score (TWS), which is expressed as a raw score ranging from 0 to 300, was used for these analyses.

### 2.2. Cognitive and Self-Report Measures

Verbal Ability: The American version of the National Adult Reading Test (AMNART) was used to measure verbal ability [61].

Emotional Intelligence: The Mayer–Salovey–Caruso Emotional Intelligence was used to assess emotional intelligence Test (MSCEIT) [62]. 

Fluid Intelligence: Fluid intelligence, comprising a variety of cognitive skills, was measured with the Raven’s Advanced Progressive Matrices (RAPM) [63]. 

Self-Compassion: The Self-Compassion Scale (SCS) was used to assess self-reported self-compassion [64]. The SCS has six subscales: Self-Kindness Items (SK), Self-Judgment Items (SJ), Common Humanity Items (CH), Isolation Items (II), Mindfulness Items (MI), and Over-Identified Items (OI).

Mindfulness: The Five Facet Mindfulness Questionnaire (FFMQ) was used to assess mindfulness [65]. The FFMQ has five subscales: Observing Items (OI), Describing Items (DI), Acting with Awareness Items (AWA), Non-Judging of Inner Experience Items (NJ), and Nonreactivity to Inner Experience Items (NRI). 

### 2.3. Amount of Practice

Lifetime hours and years of yoga or meditation practice were estimated following a structured detailed interview conducted by SWL. It should be noted that the reported number of hours are very rough estimates. However, we and others have previously found this metric to correlate with some outcome measures [52,66].

### 2.4. Statistical Analysis

The relationships among TWS and the other variables were tested with Pearson product–moment correlations. ANCOVA was performed to compare TWS of the three groups, with age, education, fluid intelligence, and verbal ability as covariates. If significant, ANCOVA were followed up by Tukey HSD post hoc tests to compare groups pair-wise [67]. Levene’s test of homogeneity of variances was used to assess equality of variances [68]. Whenever the assumption of homogeneity of variances and normality were not met, Welch’s test of equality of means was used instead of ANOVA [69]⁠. The Games–Howell test was used for the subsequent pair-wise comparisons of groups when the assumptions were not met [70]. These analyses were done with SPSS 25 (SPSS Inc., Chicago, IL, USA) and JASP (Version 0.13.1; https://jasp-stats.org (accessed on 21 July 2020)).

### 2.5. Neuroimaging

Imaging data were collected on a Siemens 1.5 Tesla Avanto MRI scanner (Erlagen, Germany) at the Martinos Center for Biomedical Imaging. Structural images were acquired using a T1-weighted magnetization prepared rapid acquisition gradient echo (MPRAGE) sequence (128 sagittal slices, slice thickness = 1.33 mm, TR = 2.73 s, TE = 3.39 ms, flip angle = 7°, field of view = 256 × 256 mm, matrix = 192 × 192 mm). Cortical reconstruction and volumetric segmentation were performed with the standard pipeline of the FreeSurfer (FS) image analysis suite (Version 5.3; http://surfer.nmr.mgh.harvard.edu/ (accessed on 21 July 2020)) [71,72,73]. During a 5-min functional resting state scan, twenty-five sagittal slices with 1 mm gap (voxel size: 3.13 mm × 3.13 mm × 5 mm) were acquired inter-leaved using a gradient echo T2∗-weighted sequence (TR = 2.5 s, TE = 40 ms, FA = 90°, field of view = 320 mm × 320 mm, matrix = 64 mm × 64 mm). 

In order to investigate the structural correlates of ego development, we correlated each subject’s TWS with their cortical thickness maps on a vertex-wise basis for the entire dataset in FreeSurfer. We used a 3-way ANOVA to determine if the correlation between TWS and cortical thickness differed between groups. Then, significant ANOVAs were followed up with between-group comparisons. We performed a clusterwise correction for multiple comparisons with a Monte Carlo simulation with a cluster-forming threshold of *p* < 0.01 and *n* = 10,000 to correct the maps showing the correlation between thickness and TWS across all groups. Age was included as a nuisance factor in all analyses. 

The resting state functional data were preprocessed using FreeSurfer and AFNI. The data were intensity normalized and registered to the anatomical scan in FreeSurfer; motion-corrected in AFNI; resampled to the left and right hemispheres on the surface and the subcortical volume in the MNI305 space (2 mm isotropic voxel size); and then smoothed to 15 mm FWHM. Based on our a priori hypotheses, the PCC was selected as our region of interest, and the PCC cluster from our structural analysis was used by selecting all vertices within this corrected surface-based cluster to create a label of the PCC. For each subject, the vertex-wise connectivity maps, or partial correlation coefficient (PCCoef) maps, were created with the PCC seed ROI-average time course as a regressor of interest. Masks of white matter signal and cerebrospinal fluid signal were defined from each subject’s FreeSurfer-parcellated structural scan and, along with motion correction parameters, were included as regressors of no interest [74]. In addition, the first 4 time points were discarded to allow for stabilization of the signal. A mask of the DMN was created by thresholding the average map at *p* < 0.01. Prior to analyzing the data, we decided to perform small volume correction (SVC) based on our a priori hypotheses; thus, we restricted our calculations to the regions included in our DMN mask. 

Group analyses were performed for the PCC connectivity maps with each subject’s TWS correlated vertex-wise with the connectivity values (z-scores) of each subject’s connectivity map to generate the final correlation maps. We first combined all subjects into a single group and correlated TWS with the connectivity maps. Next, we performed a 3-way ANOVA on the group-averaged maps. Then, pair-wise between-group t-tests were performed, followed by within-group analyses. Maps were corrected using a Monte Carlo simulation implemented in FreeSurfer with a cluster-forming threshold of *p* < 0.01 and *n* = 10,000 simulations. 

## 3. Results

Consistent with previous studies [75,76], the total weighted score (TWS) on the MAP exhibited a strong and significant relationship with educational attainment (*r* = 0.504; *p* < 0.001). As expected, there was no correlation between TWS and age, fluid intelligence, verbal ability (AMNART), or sex. There was no correlation between fluid intelligence and verbal ability. TWS correlated with FFMQ OI (*r* = 0.323; *p* = 0.037), FFMQ NRI (*r* = 0.334; *p* = 0.029), and SCS SK (*r* = 0.315; *p* = 0.037). Contemplative experience, measured as the number of lifetime hours spent practicing either mindfulness meditation or Kripalu yoga, showed a small to moderate correlation with TWS that approached significance (*r* = 0.290; *p* = 0.063). TWS did not correlate with any other variables. Contemplative experience correlated with FFMQ OI (*r* = 0.441; *p* = 0.006), total FFMQ (*r* = 0.318; *p* = 0.046), SCS SK (*r* = 0.319; *p* = 0.045), SCS MI (*r* = 0.347; *p* = 0.028), total SCS (*r* = 0.338; *p* = 0.033), and moderately but not significantly with FFMQ NRI (*r* = 0.306; *p* = 0.058). RAPM correlated with age (*r* = −0.452; *p* = 0.002), FFMQ NJ (*r* = 0.353; *p* = 0.02), and SCS CH (*r* = −0.356; *p* = 0.018).

**Figure 1:**
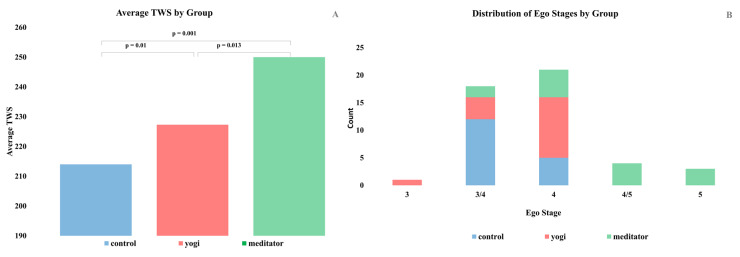
(**A**) Differences in mean TWS between groups. Mean TWS for each group is shown along with the *p*-values from the Games–Howell tests, which show that TWS is higher for meditators and yogis than controls and higher for meditators than yogis. (**B**) Distribution of stages between groups. The distribution of ego stages for the sample is shown. Stage 4 is the Conscientious stage, which is the last of the conventional stages.

In order to control for potential confounding variables, ANCOVA was performed controlling for age, education, verbal ability, and fluid intelligence. The ANCOVA revealed that there was a significant difference in mean TWS between groups when adjusting for age, education, verbal ability, and fluid intelligence (F(2,32) = 17.672, *p* < 0.001, partial η2 = 0.525; see Figure 1a). The covariates education (F(1,32) = 7.391, *p* = 0.01, partial η2 = 0.188), age (F(1,32) = 5.416), *p* = 0.026, partial η2 = 0.145), and fluid intelligence (F(1,32) = 4.332, *p* = 0.045, partial η2 = 0.119) were found to significantly adjust the association between TWS and group. According to Levene’s test, the condition of equal variances was not met (*p* = 0.022). Games–Howell post hoc tests revealed that TWS was significantly higher for meditators (250 ± 23.1, *p* < 0.001) and yogis (227.3 ± 12.2, *p* = 0.003) when compared to controls (214.0 ± 13.1). Meditators also had significantly higher mean TWS than yogis (*p* = 0.028), despite yogis having more hours (13,534) of contemplative experience than meditators (7775) on average. The distribution of stages between groups is depicted in Figure 1b. 

There were no significant group differences in age or verbal ability. There were significant differences in fluid intelligence (*F*(2,41) = 4.492, *p* = 0.017) and education (F(2,40) = 3.282, *p* = 0.048) between groups as revealed by ANOVA. Tukey post hoc tests indicated group differences in fluid intelligence (*p* = 0.013), with a significantly higher fluid intelligence for the yoga group (23.3 ± 4.8) compared to the control group (17.1 ± 6.9). Post hoc tests did not reveal any pair-wise differences in education.

**Figure 2:**
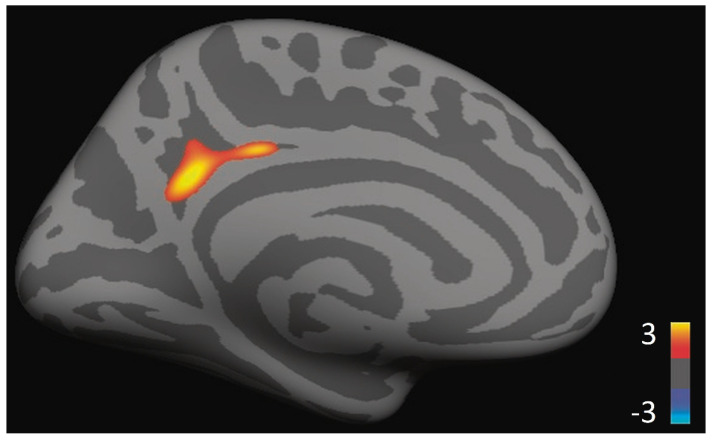
Ego development correlates with cortical thickness in the PCC/precuneus in the overall sample (*p* = 0.0237).

When the subjects were combined into one group, correcting within the DMN, a significant correlation between TWS and cortical thickness was found in a cluster overlapping the left isthmus cingulate, PCC, and precuneus (clusterwise *p*-value (CWP) = 0.0237, max = 3.082, MNI coordinates: −9.6, −51.3, 27.5, size = 356.89 mm^2^; see Figure 2). No significant correlations between TWS and cortical thickness were found in the right hemisphere. An ANOVA detected no significant differences across groups in the left or right hemisphere. Exploratory within-group analyses revealed no correlations between TWS and thickness. Exploratory whole brain analyses revealed no additional significant clusters in either hemisphere. 

**Figure 3:**
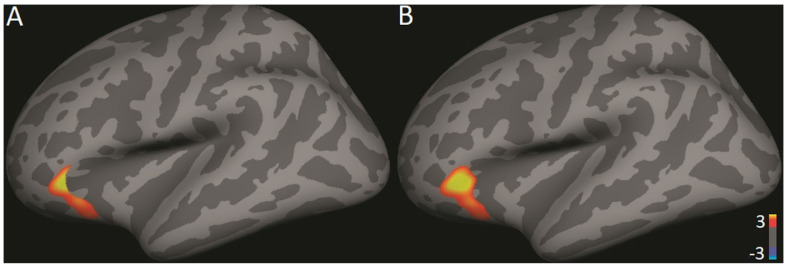
(**A**) Correcting within the DMN, TWS, and functional connectivity between the PCC/precuneus seed and the left IFC are significantly correlated (*p* = 0.0088). (**B**) The correlation between TWS and functional connectivity between the PCC/precuneus and the left IFC survived whole brain correction (*p* = 0.0269).

We conducted analyses within the DMN using the SVC method detailed above. We further conducted exploratory analyses of significant results to investigate whether the findings survived when corrected over the entire volume. When the subjects were combined into one group, analysis revealed a significant positive correlation between TWS and functional connectivity between the PCC/precuneus loci identified above and the left inferior frontal cortex (IFC; CWP = 0.0088, max = 3.697, MNI coordinates: −45.5, 33.6, −6.1, size = 471.82 mm^2^; see Figure 3a). When the subjects were combined into one group, the correlation between TWS and functional connectivity between the PCC/precuneus and the left IFC survived whole brain correction (CWP = 0.0269, max = 3.773, MNI coordinates: −43.5, 33.6, −5.8, size = 737.83 mm^2^; see Figure 3b). Thus, the exploratory whole brain analysis supported the results of the analysis that used SVC. 

**Figure 4:**
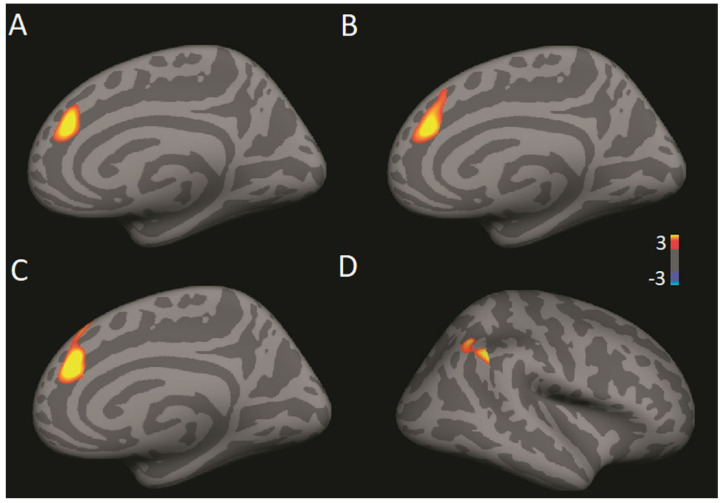
(**A**) Across all groups, there is a significant difference in the correlation between TWS and functional connectivity between the PCC/precuneus region and right (*p* = 0.0384). (**B**) Comparing meditators vs. controls, there is a significant difference in the correlation between TWS and functional connectivity between the PCC/precuneus and the right dmPFC (*p* = 0.0073). (**C**) Comparing yogis vs. controls, there is a significant difference in the correlation between TWS and functional connectivity between the PCC/precuneus and the right dmPFC (*p* = 0.0045). (**D**) Comparing yogis vs. controls, there is a significant difference in the correlation between TWS and functional connectivity between the PCC/precuneus and the right TPJ (*p* = 0.0319).

**Figure 5:**
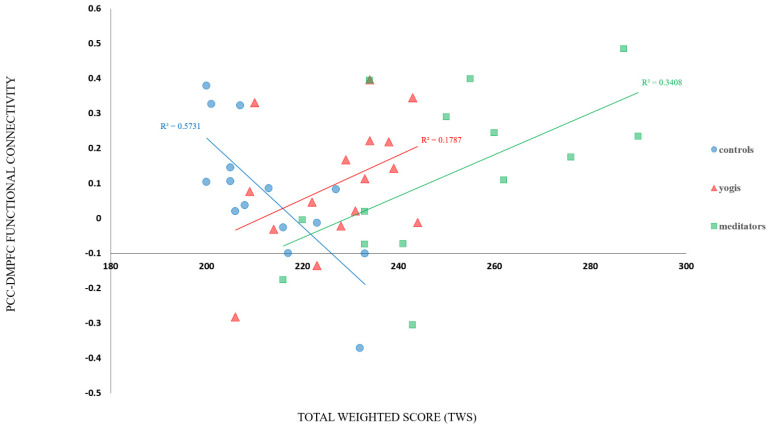
The correlation between TWS and PCC-dmPFC connectivity showing inflection points at the threshold between the Self-Conscious (3/4) and Conscientious (4) stages, indicated by a TWS of ≈220.

A GLM revealed a significant difference across groups in the correlation between TWS and functional connectivity between the PCC/precuneus region and right dmPFC (CWP = 0.0384, max = 3.668, MNI coordinates: 12.3, 36.6, 26.1, size = 349.50 mm^2^; see Figure 4a). A pair-wise contrast of meditators and controls revealed a significant difference in the correlation between TWS and functional connectivity between the PCC/precuneus and the right dmPFC for meditators compared to controls (CWP = 0.0073, max = 3.496, MNI coordinates: 13.6, 36.5, 24.5, size = 476.82 mm^2^; see Figure 4b). A pair-wise contrast of yogis and controls revealed a significant difference in the correlation between TWS and functional connectivity between the PCC/precuneus and the right dmPFC (CWP: 0.0045, max = 4.426, MNI coordinates: 10.9, 37.0, 27.2, size = 544.23; see Figure 4c) and also the right temporoparietal junction (TPJ) for yogis compared to controls (CWP = 0.0319, max = 3.350, MNI coordinates: 52.4.3, −48.6, 37.7, size = 375.69 mm^2^; see Figure 4d). A whole brain exploratory analysis indicated that for the pair-wise comparison of yogis and controls, the significant difference in the correlation between TWS and functional connectivity between the PCC/precuneus and the right TPJ survived, even with whole brain correction (CWP = 0.0257, max = 3.874, MNI coordinates: 53.7, −44.3, 40.3, size = 728.60 mm^2^). Interestingly, in the pair-wise comparisons, the correlations between TWS and functional connectivity were positive for the yoga and meditation groups but negative for the control group (Figure 5).

## 4. Discussion

The present study attempted to identify neural correlates of ego development while also providing insights into the relationship between contemplative practice and ego development. The PCC has been implicated in numerous self-related processes, including autobiographical memory, self-referential thought, first-person perspective-taking, theory of mind, and emotion [35,36,77,78]. This structure has also been linked to meditation training [57,59]. Our data suggest that the PCC plays a role in maturity development and mediates the relationship between meditative practices and maturity development. Although the exact biological mechanisms are beyond the scope of this study, there may be a positive feedback loop in which repeated practice of mindful self-awareness during meditation or yoga leads to a gradual change in the related circuits of the brain, which then allows continued growth in self-perspective. This change in self-perspective may interact with worldview to produce growth in ego development. Thus, when one’s relationship to the self changes, the self-related brain networks change as well. Likewise, when one’s relationship to the world changes, the relevant brain networks may change in response.

As previously stated, our hypotheses were focused on the DMN, since this network DMN has been consistently demonstrated to be integral to processes and perceptions related to the self. The hypotheses were that (1) TWS would be higher in subjects who meditate or practice yoga than in non-meditating matched control subjects; (2) thickness in the PCC, which is the core hub of the DMN, would correlate significantly and positively with scores on the MAP; (3) functional connectivity within the DMN would correlate with TWS; and (4) scores on the MAP would correlate with cortical thickness and functional connectivity group differences. It is important to note that in no instance is contemplative practice used as a proxy for ego development; rather, we investigate levels of ego development in each of these three groups in order to understand if there is (a) generally a relationship between ego development and the brain and (b) if there is a relationship between ego development and contemplative practice.

Our results revealed there was a statistically significant difference in TWS between the groups when controlling for age, education, verbal ability, and fluid intelligence, with a large effect size. As hypothesized, both the meditation and yoga practitioners scored significantly higher than the controls on the MAP. The majority of the control group was at the Self-Conscious stage (3/4), while the majority of yogis and meditators were at or above the Conscientious stage (4). We only identified individuals at the post-conventional Individualist stage (4/5) and Autonomous stage (5) in the meditation group. Typically, only 15–20% of the US population scores in the post-conventional range [4], yet fully half of our sample of meditators were at a post-conventional stage. Cook-Greuter emphasizes that at these stages adults begin to realize that the “meaning of things depends... on one’s personal perspective and interpretation of them” [4]. In particular, those at the Autonomous stage are capable of “integrating many disparate parts of themselves”, of integrating their varied sub-identities [4]. 

The between-group differences in ego development were not accounted for by age, sex, verbal ability, or fluid intelligence, which is consistent with previous findings [5]. The yoga group was found to have higher fluid intelligence than the control group, which may be related to yoga, which involves both physical movement and breathing exercises, preserving intelligence in aging [26,27]. Alternately, this finding could be due to individuals with higher fluid intelligence, which correlates with socioeconomic status, being attracted to yoga due to reasons of health or status [79,80]. Two subscales of the self-report measure of mindfulness, specifically the “observing” and “non-reactivity” subscales of the FFMQ, were positively correlated with ego development, which is consistent with individuals at higher ego development stages being more capable of observing and understanding their inner processes with less judgment [2]. In addition, we found that the self-kindness subscale of the self-compassion scale correlates with ego development, which is in agreement with research showing that higher ego development is related to emotional security and intimacy [19] and increasing compassion and identification with all life [4]. 

The between-group difference in ego development is marginally correlated with time spent practicing meditation or yoga in the two contemplative groups, which is consistent with the conceptual and empirical linkage between ego development and contemplative practice. Interestingly, although the yogis had on average twice as many lifetime hours of practice as the meditators, their scores on the MAP were significantly lower. One possible explanation could be that the Buddhist tradition includes specific practices to foster compassion and understanding for others (e.g., metta) and places a strong emphasis on morality, each of which are important components of higher stages of ego development. Alternatively, individuals who are at higher ego stages, or who were more likely to advance to higher stages, chose to practice meditation rather than yoga. Of note, these same yoga practitioners scored higher than the meditators on a measure of fluid intelligence and also on a metric of brain network integrity [26], suggesting that these traditions may differentially impact various neural processes.

In terms of brain gray matter structure, the only locus identified was a sub-region of the posterior cingulate cortex and precuneus (PCC/precuneus), which comprise the primary node of the default mode network [81]. The DMN network is thought to integrate experience [82], and is active in creating an integrated representation of the self. The PCC/precuneus are also implicated in integration of information, self-referential thought, empathy, and control of external and internal awareness [28,30,83] as well as both first-person and third-person perspective taking [84,85]. Each of these cognitive functions is related to higher stages of ego development [2], and so the correlation between PCC/precuneus cortical thickness and TWS is consistent with ego development theory. In terms of functional connectivity, we identified a strong correlation between ego development and connectivity between the PCC/precuneus and the left IFC when data from all groups were combined. A recent meta-analysis identified three sub-regions within the IFC that are responsible for the following: affective empathy, semantic and phonological processing, and working memory [86]. Our cluster appears to span the sub-regions implicated in empathy and semantic processing. 

Language and narrative play a primary role in the creation, maintenance, and integration of the self [87,88,89,90,91] and are at the foundation of ego development theory as a tool for understanding and constructing reality [2]. The left IFC is also part of the DMN [92], and it plays a role in narrative comprehension and production, particularly inference of implied events and inhibition of self-referential thought [93,94]⁠. In addition, recent research suggests that the left IFC participates in both the evaluation and generation of creative or original ideas; specifically, inhibited activation results in higher creativity, and increased activity is associated with evaluation [95]. The dmPFC, another node of the DMN, is also active in processing the coherence of a story [96]. Brain research has also identified a primarily left-lateralized network involved in the production of narrative and a bilateral network involved in the understanding of narrative; regions in these networks include the PCC, TPJ, dmPFC, and IFC [97,98,99,100]—the same networks we identified in the present study. The neural circuits found in the present study may integrate information about the self, experience, and personal narratives [82] in order to facilitate the complex processing associated with higher stages of ego development. Interestingly, connectivity between the PCC and right dmPFC, which is involved primarily in narrative comprehension, correlated with TWS in only the meditation and yoga groups, while PCC connectivity with the IFG, which is more involved with narrative production, correlated with TWS when all three groups were combined. This finding gives additional weight to the notion that contemplative practice supports ego development by progressively strengthening the neural circuits necessary to organize and comprehend an increasingly complex personal narrative.

In addition, we found that the relationship between ego development and PCC-TPJ connectivity was greater for the contemplative practitioners compared to the control group. The TPJ, similarly to the IFC, has been implicated in emotion, empathy, and theory of mind [101,102,103], which are traits that are associated with both higher TWS and regular meditation practice. The dmPFC is active in processing both first-person perspective and third-person perspective [84,85], and it has also been linked to theory of mind [104,105]. 

The construction of complex narrative and interpersonal understanding requires an integration of empathy and language that the increased connectivity among these regions may represent, and this connectivity may play a role in performance on the MAP. Recent research has found increased connectivity between the PCC and IFC during Buddhist loving-kindness meditation [106]⁠, which may lead to long-lasting increases in functional connectivity between these regions during rest. The PCC has also been implicated in various functional activation studies of meditation [56,107], and our group has found increases in PCC/precuneus and TPJ gray matter density after 8 weeks of meditation training [59]. We hypothesize that meditation-related changes in gray matter may underlie changes in structural and functional connectivity between these regions, which could in turn contribute to growth in ego development. Our results may indicate that the PCC functions together with the dmPFC to maintain coherence of the individual’s personal narrative, providing stability and the ability to organize their life meaningfully. Thus, it may be possible that contemplative practice supports ego development by progressively strengthening the neural circuits necessary to organize an increasingly complex personal narrative, which manifests as Cook-Greuter’s “increasing levels of embrace” [4]. Further research is needed that specifically and rigorously tackles these questions.

Interestingly, our results indicated that the correlation between ego development and PCC-TPJ and PCC-dmPFC connectivity was positive for contemplative practitioners but negative for control participants. Furthermore, the point at which the fit lines intersected was at the TWS boundary between the Self-Conscious (3/4) and Conscientious (4) stages. Unfortunately, because the controls are mostly at stages 3 and 3/4 and the contemplative practitioners are at stages 4 and above, we cannot know if the difference in slope is due to group effects of contemplative practice or whether it is a true difference between the stages that is reflected in the brain. Further research will be needed to determine if the slope for participants without meditation experience also reverses at this boundary. It is interesting to note that a primary distinction between levels 3/4 and 4 is the ability to objectively take another’s perspective, which is a function strongly associated with these DMN nodes. 

The present study is limited by the small sample size and the inclusion of only middle-aged subjects, which especially constrains the relevance and generalization of the data to the general population. Future research beyond this pilot study should include large samples of healthy adult control subjects as well as large samples of contemplative practitioners. In addition, due to the cross-sectional nature of the study, we cannot make causal inferences regarding the interaction between meditation, brain structure and function, and ego development. The observed association between ego development and contemplative practice as well as its moderate but non-significant correlation with hours of contemplative practice may be due to regular contemplative practice facilitating the development of the self, or it may be that those with high initial levels of ego development simply gravitate toward regular contemplative practice. Furthermore, we are unable to examine brain structure and function at the highest stages of ego development, which comprises less than 2% of the US population. Although research on the sustainable advancement to higher stages of ego development and the mechanisms or processes that may facilitate this progression is scarce, there is some evidence that ego development can be developed or catalyzed through training programs [108,109,110,111,112] and transcendental meditation [25]. Given that other researchers have criticized the lack of an explanatory mechanism for growth in ego development, and Loevinger herself recognized that “there is no generally accepted theory of what accounts for progress in ego development”, clarifying a possible neurological basis for progression may aid in the development of more accurate theories of adult development [113,114].

## Figures and Tables

**Table 1:** Demographics and variables.

	Control	Yoga	Meditation	*p*
*Demographic variables*				
Age, years	52.5 (10.1)	49.4 (7.8)	54.8 (8.3)	0.25
Education, years	16.9 (2.1)	17.1 (2.5)	19.0 (2.6)	**0.048**
Sex (M/F)	7/9	5/11	5/9	0.76
Race (white/non-white)	17/0	16/0	14/0	1.00
RAPM	17.1 (6.9)	23.3 (4.8)	20.6 (4.8)	**0.017**
AMNART	119.4 (9.0)	123.3 (4.7)	125.2 (5.2)	0.071
TWS	214.0 (13.1)	227.3 (12.2)	250.0 (23.1)	**<0.001**
Amount of practice, hours	0 (0)	13,534.4 (9949.8)	7774.8 (6237.4)	0.188
Amount of practice, years	0 (0)	18.3 (9.6)	15.8 (7.6)	0.744
MSCEIT total	95.2 (15.6)	95.2 (10.4)	104.2 (8.2)	0.159
MSCEIT strategic	92.1 (10.4)	95.6 (8.4)	99.8 (7.0)	0.133
MSCEIT experiential	100.4 (19.5)	95.9 (11.3)	106.7 (10.0)	0.248
SCS total	3.4 (0.76)	3.8 (0.42)	3.9 (0.44)	**0.03**
SCE SJ	2.7 (0.98)	2.4 (0.57)	2.4 (0.68)	0.431
SCS OI	2.4 (1.0)	2.3 (0.58)	2.3 (0.42)	0.946
SCS II	2.6 (0.95)	2.1 (0.68)	2.1 (0.61)	0.121
SCS CH	3.1 (1.0)	3.5 (0.87)	3.9 (0.81)	0.072
SCS SK	3.3 (0.87)	4.1 (0.58)	4.1 (0.56)	*0.002*
SCS MI	3.6 (0.78)	4.1 (0.53)	4.3 (0.53)	*0.012*
FFMQ total	17.0 (3.1)	19.8 (2.6)	19.5 (2.5)	**0.016**
FFMQ OI	3.1 (0.70)	4.1 (0.45)	4.0 (0.32)	**<0.001**
FFMQ DI	3.7 (0.75)	4.1 (0.62)	3.9 (0.68)	0.295
FFMQ NJ	4.2 (0.76)	4.3 (0.58)	4.2 (0.75)	0.862
FFMQ NRI	3.1 (0.75)	3.8 (0.44)	4.1 (0.63)	**<0.001**
FFMQ AWA	3.7 (1.0)	3.7 (0.70)	3.6 (0.55)	0.903

## Data Availability

The data that support the findings of this study are available on request from the corresponding author. The data are not publicly available due to their containing information that could compromise the privacy of research participants.
